# Effects of bone types, particle sizes, and gamma irradiation doses in feline demineralized freeze-dried bone allograft

**DOI:** 10.14202/vetworld.2020.1536-1543

**Published:** 2020-08-10

**Authors:** Frizky Amelia, Basril Abbas, Darmawan Darwis, Sri Estuningsih, Deni Noviana

**Affiliations:** 1 Program Study of Animal Biomedical Science, Graduate School of IPB University, Bogor, Jawa Barat 16680, Indonesia; 2 Diagnostic Imaging Center, Veterinary Teaching Hospital, Faculty of Veterinary Medicine IPB University, Bogor, Jawa Barat 16680, Indonesia; 3 Centre for Isotopes and Radiation Application, National Nuclear Energy Agency (BATAN), Jakarta Selatan, DKI Jakarta 12440, Indonesia; 4 Department of Clinic Reproduction and Pathology, Faculty of Veterinary Medicine IPB University, Bogor, Jawa Barat 16680, Indonesia

**Keywords:** bone type, feline bone allograft, gamma irradiation, *in vitro* study, particle size

## Abstract

**Background and Aim:**

Fracture cases significantly increase recently, demanding high quality of bone graft materials. This research aimed to evaluate the effects of bone types, particle sizes, and gamma irradiation doses on morphological performance and cell viability of feline demineralized freeze-dried bone allograft (DFDBA) through an *in vitro* study.

**Materials and Methods:**

Feline DFDBA derived from feline cortical and cancellous long bones was processed into four different sizes: Group A (larger than 1000 µm), B (841-1000 µm), C (420-840 µm), and D (250-419 µm) for each type of bones. The materials were then irradiated with two doses of gamma rays, 15 and 25 kGy, resulting in 16 variants of feline DFDBA. The surfaces of each material were then observed with the scanning electron microscope (SEM). The *in vitro* evaluation of feline DFDBA was then performed using 3-(4,5-dimethythiazol-2)-2,5-diphenyltetrazolium bromide (MTT) assay with calf pulmonary artery endothelial cells.

**Results:**

The MTT assay results showed that the lowest inhibition rate (14.67±9.17 %) achieved by feline DFDBA in Group A derived from cortical bones irradiated with 15 kGy. Group D generally showed high inhibition rate in both cancellous and cortical bones, irradiated with either 15 or 25 kGy. The SEM results showed that cancellous and cortical bones have numerous macropores and micropores structure in 170× and 3000×, respectively.

**Conclusion:**

The material derived from cortical bones in Group A (larger than 1000 µm in particle size) irradiated with 15 kGy is the best candidate for further development due to its abundance of micropores structure and ability in preserving the living cells.

## Introduction

Bone is the second most common organ transplanted after blood [1]. Bone grafting is a bone replacing technique by donor bone or bone-like material in a gap or around the broken bone through the surgical procedure on a recipient that can stimulate bone healing processes [1,2]. There are several types of bone graft with their own advantages and disadvantages [3,4]. Autograft, isograft, allograft, and xenograft are well known in both human and veterinary medicine [1-3,5-7]. Each type of available bone graft possesses a different level of osteogenic, osteoinductive, and osteoconductive properties [1,3,8]. Therefore, bone graft material should be chosen carefully depending on the need of each orthopedic abnormalities, the availability of material, financial consideration, as well as the surgeon’s preferences [1,2,9].

Bone allografting is a grafting technique developed using materials derived from other animals that are not identical but comes from the same species [2-6,8]. The allografting technique has the risk of immune rejection and pathogen transmission to the recipient [3,5,6,10,11]. However, the allograft technique also provides an excellent alternative to autograft. It has the advantages of higher availability in terms of quantity compared to autograft and lower rejection reaction compared to xenograft while also can be done in a single surgery procedure [3,5,6]. Allograft can also avoid sacrificing the normal structure of the patient’s anatomy, reduce the need for prolonged hospital care and cost, and it also allows manipulation of material’s size and shape [6]. A proper preparation and sterilization techniques of bone allograft material are required to achieve a good bone healing process afterwards [6]. The synthesis and sterilization processes can reduce the antigenicity and the risk of disease transmission [3,5,6,10,12-15]. Due to the lack of research, the description of the combined effect of bone type, particle size, and irradiation technique and doses and their interaction on cell viability of bone allograft materials are not well described, especially for materials derived from and purposely used in an animal.

Bone graft material cannot be used or applied directly right after production unless they have been tested in several studies ranging from material characteristics, *in vitro* testing, and *in vivo* testing [4,16]. *In vitro* study is important in material development, especially in detecting wide spectrum of unspecific mechanisms, and effects of xenobiotics before the material are tested in a more complex environment of living animals or *in vivo* study [8,16]. Furthermore, *in vitro* study can be used to measure the concentration when a substance starts to damage components, structures, or biochemical pathways within the cells [16].

In this study, the feline demineralized freeze-dried bone allograft (DFDBA) as bone filler materials was developed from feline cadaver bones. A total of 16 variants of feline DFDBA were successfully produced with a combination of two different bone types (cancellous and cortical bones), four particle sizes (ranging from 250 to larger than 1000 µm), and two irradiation doses (15 and 25 kGy). The materials produced then underwent an *in vitro* test using the 3-(4,5-dimethythiazol-2)-2,5-diphenyltetrazolium bromide (MTT) assay to evaluate its ability in preserving living cells.

## Materials and Methods

### Ethical approval

There is no living animal involved in this *in vitro* study. Nevertheless, an ethical clearance from the Institutional Animal Care and Use Committee of Institute Pertanian Bogor (IPB) University was given for our whole project (including the *in vivo* study) with approval number: 100-2018 IPB.

### Study period and location

The synthesis and sterilization of bone allograft materials proceeded at the Biomaterial Laboratory, Centre for Isotopes and Radiation Application (CIRA) - National Nuclear Energy Agency (BATAN) from September 2016 to June 2017. The *in vitro* study took place at the Primate Research Centre (PSSP) - IPB University in August 2017. Meanwhile, the materials characterization proceeded at the Center for Science and Advanced Material Technology (PSTBM) BATAN in November 2017.

### Synthesis, sterilization, and characterization

Feline DFDBA is synthesized and sterilized at Biomaterial Laboratory, Radiation Processing Division, Centre for Isotopes and Radiation Application (CIRA) – National Nuclear Energy Agency (BATAN) of Indonesia. Sterilization of feline DFDBA was carried out using gamma rays from Co-60 source (Gamma Cell 220 Cobalt 60 Irradiator MDS^®^, Canada). The characterization process of feline DFDBA was carried out using a scanning electron microscope-energy dispersive X-ray spectroscopy or SEM-EDS (JEOL JSM-6510LA^®^, Japan) at the Center for Science and Advanced Material Technology (PSTBM) BATAN.

### Material preparation

The raw materials used for feline DFDBA are the cancellous and cortical region of feline cadaveric long bones obtained from several clinics and animal hospitals in Jakarta and Bogor cities. The feline cadaver used in this study has been approved by its owner and or clinic with a consent letter. The medical records were noted to make sure that the donor’s bones were not afflicted by any infectious diseases.

The allograft materials were made following the protocol at the Biomaterial Laboratory CIRA BATAN [14]. Each type of bones obtained was demineralized using hydrochloric acid 0.6 M solution and then lyophilized by freeze dryer until the water content lowered to <10 % to produce the feline DFDBA. It was then ground using bone mills to process the bones into granules. The bone then filtered using three different sizes of 20, 40, and 60 mesh filters to produce four different sizes, that is, larger than 1000 µm (Group A), 841-1000 µm (B), 420-840 µm (C), and 250-419 µm (D). The feline DFDBAs were sterilized using gamma-ray with 15 and 25 kGy irradiation doses. Using these procedures, 16 feline DFDBA variants were produced.

The morphological performance of feline DFDBAs was analyzed using the SEM-EDS. Particles of feline DFDBA were fixed on stubs using carbon tape or adhesive containing powdered graphite and sputter-coated with gold in an ion coater. The particle size and morphology of the materials were examined under vacuum with SEM using 20.0 kV and 1.00000 nA probe.

### *In vitro* study with MTT assay

The *in vitro* testing of feline DFDBA was conducted at Primate Research Center IPB University, Indonesia. The culture of Calf Pulmonary Artery Endothelial (CPAE) cells (CPAE, ATTC® CCL-209TM, USA) was grown in media consisting of Dulbecco’s Modified Eagle’s growth Medium (D-MEM, Sigma-Aldrich®, USA), fetal bovine serum 20%, and a combination of antibiotics, that is, penicillin 100 U/mL-streptomycin 100 ug/mL. The atmosphere was set at 5% carbon dioxide or CO_2_ [16]. The MTT assay testing was done in duplicate. Cells were grown with a concentration of 4.5×10^3^ cells per 100 µL media. The bone graft material was added after the cell reaches 50% confluence in 24 h. The MTT test was conducted on the 3^rd^ day by adding MTT (5 mg/mL) of 10 µL per well, then incubated for 4 h at 37°C [16]. Formazan crystals were dissolved in ethanol. The reading of the absorbance value was carried out at a wavelength of 595 nm using a microplate reader (Bio-Rad^®^, USA) against a reference well-containing cell without bone graft produced. Absorbance correlates linearly with cell number over a specific optical density range of 0.2-1.0 [16].

### Statistical analysis

The data achieved from the *in vitro* study were analyzed using a complete randomized design with analysis of variance where p<0.05 was considered statistically significant. The significant differences were then evaluated with Duncan’s test. The mean values±standard deviation of inhibition rate was then displayed and presented descriptively.

## Results

In this study, 16 variants of feline DFDBA were produced with a combination of two types of bones, four different ranges of particle sizes, and two irradiation doses. The results of the MTT assay showed that only 10 bone graft variants produced have an inhibition rate of less than 50% (Table-1). The other six variants of feline DFBDA showed an inhibition rate higher than 60%. The variants are feline DFDBA cancellous in Group B both irradiated with 15 kGy (73.62±5.15 %) and 25 kGy (63.71±8.46%), and all variants in Group D regardless the bone type and irradiation doses applied (Table-1).

**Table-1:** The inhibition rate of 16 variants feline demineralized freeze-dried bone allograft produced.

Bone graft type	Irradiation dose (kGy)	Particle Size (µm)			

		A	B	C	D
			
		(>1000)	(841-1000)	(420-840)	(250-419)
DFDBA Cancellous	15	35.25±6.66^cd^	73.62±5.15^a^	32.27±6.29^cde^	81.93±1.09^a^
	25	27.95±15.90^de^	63.71±8.46^ab^	36.91±5.15^cd^	78.01±5.93^a^
DFDBA Cortical	15	14.67±9.17^e^*	19.25±8.99^de^	37.83±6.13^cd^	82.10±0.49^a^
	25	21.86±12.28^de^	26.30±14.40^de^	36.61±10.66^cd^	82.06±3.35^a^

* The feline DFDBA derived from the cortical bone in Group A is significantly different by ANOVA (p<0.05) and Duncan test and considered as the best material based on its lowest inhibition rate (showed by superscript “e”). The superscript alphabets list the inhibition rates from each material start from the highest “a” to lowest “e” inhibition rate.

**Figure-1:**
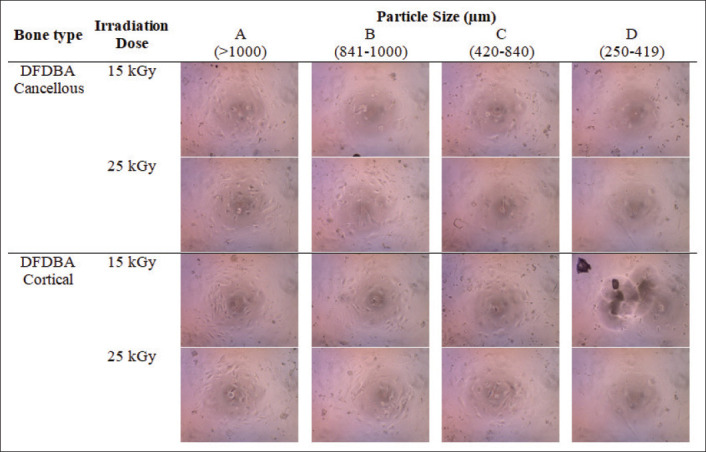
3-(4,5-Dimethythiazol-2)-2,5-diphenyltetrazolium bromide assay result of 16 variants of feline demineralized freeze-dried bone allograft observed in 40× with a light microscope (CX23 Olympus^®^, Japan).

**Figure-2:**
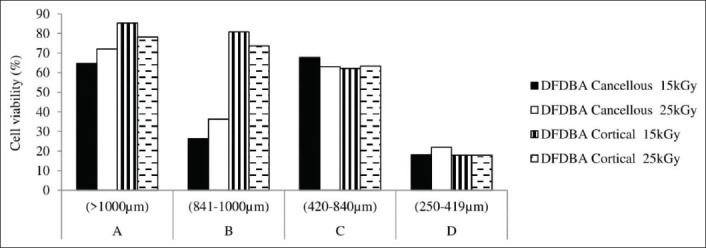
The cell viability of 16 variants of feline demineralized freeze-dried bone allograft produced.

While observing cell viability (by reducing 100% with inhibition rate percentage), feline DFDBA in Group A (larger than 1000 µm) derived from cortical bone irradiated with 15 kGy can be observed good in preserving the living cells (85.34%). On the other hand, Group D generally showed less number of living cells within the well compared to other particle sizes groups. Figure-1 shows the microscopic findings of the *in vitro* testing, while Figure-2 shows the comparison of cell viability among the materials produced.

In this study, it can be seen from Table-1 and Figure-2 that generally feline DFDBA in Group A tends to have the lowest inhibition rate and highest cell viability than any other group. On the contrary, feline DFDBA in Group D showed the highest inhibition rate and lowest cell viability.

**Figure-3:**
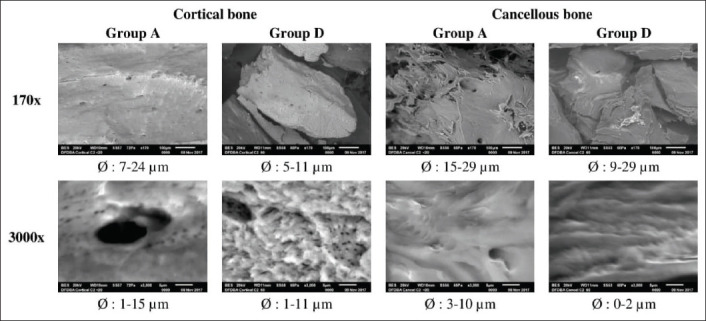
Scanning electron microscopes image in 170× and 3000× of feline demineralized freeze-dried bone allograft which showed highest cell viability (Group A) and lowest cell viability (Group D). Scale bar indicated 100 µm width in 170× and 5 µm width in 3000×.

Looking closer into the material structure and surface using SEM, it can be seen that all materials were in an irregular shape with various size of micropores. The micropore diameter of feline DFDBA in Group A is 7-24 µm (cortical) and 15-29 µm (cancellous) in 170×. Meanwhile, the feline DFDBA in Group D has the diameter of micropores being 5-11 µm (cortical) and 9-29 µm (cancellous) in 170x. From 170× with SEM, the cancellous bone is more likely to have larger micropores than cortical bone. However, in 3000×, it can be seen clearly that material in Group A has larger micropores compared to materials in Group D, regardless of bone type origin (Figure-3).

## Discussion

Freeze-dried bone allografts are the most common material used as allogenic grafts in bone transplantation [17]. In this study, the DFDBAs for cat patient (feline DFDBA) were made and evaluated using MTT assay, which is one of the *in vitro* testing techniques that can be used to identify the cytotoxicity of materials and measure the cell viability and proliferation in response to external factors [16,18]. This assay will detect the reduction of tetrazolium salt to examine the cell proliferation in the media after being added with the feline DFDBA produced. Yellow tetrazolium MTT is reduced by mitochondrial succinate dehydrogenase on entering cells by turning it into a water-soluble and dark purple formazan product [18]. Unable to pass cell membrane, the product accumulates within the cell [16]. Due to the accumulation, the cell then will be solubilized and liberated with organic solvents, isopropanol or ethanol, and the formazan reagent. Dissolved cells can be quantified by spectrophotometry through colorimetry [16,18]. The ability of cells to reduce MTT provides an indication of mitochondrial integrity and activity, which can be interpreted as a measure of cell viability because the MTT reduction can only occur in metabolically active cells [16,18].

### Endothelial cells

In this study, the CPAE cells were used in the MTT assay because the endothelial cells play a big role in bone healing process. Endothelial cells synthesize and secrete physiologically important molecules into the blood and/or the subendothelial extracellular matrix [19,20]. These molecules participate in the formation of platelet and fibrin thrombi and contribute to the antithrombotic properties of the endothelium, which are prostacyclin, thrombomodulin, and heparan sulfate [19]. Endothelial cells also synthesize and secrete plasminogen activators and inhibitors, source of molecules regulating the growth of other cells [19,20]. They synthesize angiotensin-converting enzyme and bind lipoproteins and hormones [19,21]. Capillaries invade the differentiating mesenchymal zone during intramembranous ossification, whereas in endochondral ossification, the infiltrating vasculature was recruited by hypertrophic chondrocytes [20]. Initial vascularization is followed by an invasion of osteoclasts and osteoblasts, with coordinated resorption of hypertrophic cartilage and subsequent mineralization of the extracellular matrix as well as bone formation [20,21]. Hence, vascularization occurs from the center of ossification toward the growth plate and determines the rate of bone ossification [22]. Finally, they are the target for, and participant in, immune reactions. Due to this, endothelial cells constitute not only the first barrier between the blood and the extravascular space but also serve as a source of molecules influencing the structural and functional integrity of the circulation system [19-22].

### The effect of bone types

From the MTT results of this study, it can be seen that there is a correlation between bone types, particle sizes, and irradiation doses in preserving the living cells. Figure-2 shows that from 16 variants of feline DFDBA, the material made from cortical bone has more stable results than cancellous bone in comparison to their particle sizes. Meanwhile, the irradiation doses showed more variations if combined with its bone type and particle sizes.

Several studies stated that cancellous bone has a higher osteogenic activity than cortical bone due to its porosity [1,23]. It is seen in contrast with the current result of our study (Figure-2), which showed that cortical bone generally has higher viability than cancellous bone for Groups A and B in both irradiation doses. From Figure-2, it can be observed that in cortical bone, particle size plays an important role as the cortical bone viability decreased as the particle size went smaller, regardless of the irradiation doses given to the materials. However, in cancellous bone, different patterns appeared. The cell viability in cancellous bone is higher in Groups A and C, but lower in Groups B and D. This indicated that for cancellous bone, specific particle size range needs to be put into consideration. These findings were supported by Hangody [9] who stated that each type of tendon grafts has different initial biomechanical properties and reacts differently to material preparation and sterilization processes.

This study demonstrated that each bone type showed specific criteria and can be used in different cases and need. Cancellous bone has 30-90% porosity due to its spongy structure which makes it a good candidate as a filler material in general because it can induce the formation of new bone cells by osteoblast and osteoclast [1,23]. Cortical bone with 5-30% porosity can be used to fill the gap by the extremities because it can provide strength and structural stability [23].

### The effect of particle sizes

Some alloplastic, ceramic-like materials sieved with 40-60 or 40-100 mesh filters, or about 250-425 µm or 100-425 µm in diameter were commercially produced [17]. Bigger materials were uncommon for general clinical use. In this study, the particle size is predetermined by milling process which ground the bone and sieved the particle using 20, 40, and 60 mesh filters to produce bone granules, that is, larger than 1000 µm, 841-1000 µm, 420-840 µm, and 250-419 µm in diameter, respectively.

The particle size effect on the cell viability in Figure-2 showed that both cortical and cancellous bones have the highest cell viability in Group A (larger than 1000 µm) and the lowest in Group D (250-419 µm). The possible cause for this was, in the same size of microplate wells, materials in Group A provide a larger gap between particles compared to materials in Group D where the gap between particles is smaller. This is especially shown by feline DFDBA derived from the cancellous bone (Figure-3). When the material sizes are too small, the well will be densely filled and may not provide enough inter particular space or large enough macropores to allow the migration and ingrowth of the cells. These findings are supported by previous studies which stated that large bone graft particles (1000-2000 µm) provide the best preservation of total bone volume and bone height up to 8 weeks after grafting in an animal vertical augmentation model than small particle (150-400 µm) and a mixture of both sizes [24].

In contrast, Wang *et al*. [25] reported that a microstructured biphasic calcium phosphate ceramic with particle size of 212-300 µm, 106-212 µm, and 45-106 µm showed abundant bone formation in the muscle of dogs, while material smaller than 45 µm in size showed no bone formation. The particle size range of 212-300 µm was within the same range as our feline DFDBA in Group D (250-419 µm), which showed the opposite result. Some articles show that smaller particles were more osteogenic with higher osteoinductive and osteoconductive activities [17,25]. Furthermore, research by Hruschka *et al*. [26] stated that the agglomeration of large particles causes less newly formed bone tissue to be obtained within defect site due to less space available for the new bone formation.

Further observation on the bone type of feline DFDBA in Group A, which shows the highest cell viability in general, revealed that the material derived from cancellous bone has abundant macroporous structures among the gap between particles in 170× SEM magnification (Figure-3). However, on 3000×, it can be seen clearly that the feline DFDBA derived from cortical bone has abundant small micropores in Group D and large micropores in Group A. The macropore and micropore structures of the feline DFDBA materials can act as a scaffold exhibiting osteoconductive property which supports media infiltration and ingrowth of the CPAE cells as seen in this *in vitro* study.

These results showed that there is an optimal particle size range for each bone type of feline DFDBA produced for osteoinduction and osteoconduction properties. As for feline DFDBA derived from cortical bone, the suggested size range is similar to Groups A, B, and C (range from 420 to larger than 1000 µm), respectively. Meanwhile, for feline DFDBA derived from the cancellous bone, it is suggested to be produced with a size similar to Group A (larger than 1000 µm) and C (420-840 µm).

### The effect of irradiation doses

Gamma irradiation is often used to sterilize the materials to decrease infection risk and disease transmission by inactivating bacteria, fungal spores, and viruses [10-13,15,27-31]. Some studies showed that the pathogen inactivation of gamma irradiation is dose-dependent [30,32]. Viruses are more resistant to radiation than bacterial spores [31]. The lower dose of gamma irradiation, ranging from 4 to 18 kGy, can be used to sterilize material from bacteria, but it requires a higher dose of 30-50 kGy to kill viruses, parasite, and helminth [30-32].

Gamma irradiation can also affect the mechanical and biological properties of bone allografts during the sterilization process by changing the molecular structure, for example, biological components such as cytokines, chemokines, and growth factors [10,27,31]. The irradiation process can also degrade the collagen fibers in the bone matrix which causes the bone to turn brittle. This reduces the tensile fatigue life, strength, and stiffness of bone graft material [10-12,27,28,31]. These effects are dose-dependent and tend to depend on the bone type of allograft materials [10,28,31].

In this study, two irradiation doses of 15 and 25 kGy were used to compare their effect on the inhibition rate of feline DFDBA materials through the MTT assay. The 15 kGy was chosen as the minimum irradiation dose to sterilized lyophilized (freeze-dried) bone graft material [29]. Meanwhile, the 25 kGy was chosen as the most common irradiation dose used which is also officially declared as the minimum dose for sterilizing bone allografts decided by the International Atomic Energy Agency and American Association of Tissue Banks [28,33]. The protocols of American tissue banks showed that the sterilization dose can be varied between 10 and 35 kGy [13]. This variation is also due to a wide range of confounding variables and individual decisions by tissue banks [10,33].

In Figure-2, it can be seen that at a dose of 15 kGy, the feline DFDBA derived from the cortical bone in Groups A and B and cancellous bone in Group C have higher cell viability compared to the same bone type and particle size irradiated at a dose of 25 kGy. These findings can be explained by the previous biomechanical study which demonstrated that at doses higher than 20 kGy, the bone matrix proteins are destroyed and their mechanical properties are compromised [10]. In contrast, research by Yanke *et al*. [11] showed that a low dose of 1.0-1.2 Mrad (equal to 10-12 kGy) gamma irradiation can reduce the bone-patellar tendon-bone allograft material by 20%.

Interestingly, it can also be observed in Figure-2 that the feline DFDBA derived from a cancellous bone in Groups A, B, and D and cortical bone in Group C irradiated with 25 kGy has higher cell viability than the same materials irradiated with 15 kGy. Moreover, based on the bone type of feline DFDBA in Table-1 and Figure-2, material derived from cortical bone generally has higher cell viability at 15 kGy irradiation dose. Meanwhile, the feline DFDBA derived from cancellous bone tends to have stable high cell viability at an irradiation dose of 25 kGy. This is indicated that each type of bone allograft requires different irradiation doses to preserve its mechanical and biological properties. Similar finding was also stated by Hangody (2016) in his thesis that each type of tendon graft has different initial biomechanical properties and each of them also reacts differently to gamma irradiation [9].

A previous study by Nguyen *et al*. [10] and Akkus and Belaney [28] also stated that the mechanical properties of bone allograft may decrease when the gamma dose is increased above 25 kGy for cortical bone and 60 kGy for cancellous bone. A higher dose of 25 kGy reduces the bone strength by 20-30%, and every further increase in 5 kGy will induce an additional 25% in strength reduction [31]. Another research by Aguila *et al*. [15] mentioned that gamma irradiation with 1.5-2.5 Mrad (equal to 15-25 kGy) does not cause major alterations in biomechanical properties of tendon and provide a suitable amount of sterilization for ligament reconstruction. This indicates that the efficacy and side effects of dose variations are still debatable, mainly about the guarantee level of sterility while at the same time trying to minimalize the negative biological effects of gamma irradiation [15].

Nevertheless, gamma irradiation also exhibits a positive impact other than the assurance of material sterility. A study by Tufekci *et al*. [7] revealed that at 30 kGy irradiation on the equine cortical bone under high-speed loading showed significantly higher modulus of elasticity (45%), ultimate strength (24%), and toughness (26%) than those of non-irradiated bone. Moreover, another study also reported that there is no significant difference in osteointegration between irradiated and non-irradiated bone grafts used in patients with revision hip arthroplasty [34]. Thus, further exploration of prospective techniques in reducing the negative impact of gamma irradiation or preserving the mechanical and biological properties of bone allograft material is needed.

From the result of our study and its comparison with other findings, it seems that the irradiation dose of 15 and 25 kGy can be put into consideration as the suggested dosage for sterilization process of feline DFDBA derived from cortical and cancellous bones, respectively, with minimum alteration of mechanical and biological properties. Regardless of the positive and negative impact of gamma irradiation, this technique is still believed to be an effective method to sterilize and ensure the safety of biological tissue allografts [6,35].

Hopefully, these findings will help practitioners by providing some basic information on the effect of bone types, particle sizes, and gamma irradiation doses of feline DFDBA. With recent studies showing various results for different bone graft materials, information from this study could provide a rationale for choosing bone type, particle sizes, and irradiation doses with the assumption that other factors may be equal.

## Conclusion

From these results, it can be concluded that the viability and growth of CPAE cells were influenced by the bone type, particle size, and irradiation dose of feline DFDBA materials. Among the 16 variants of feline DFDBA, the material derived from the cortical bone in Group A (larger than 1000 µm) irradiated with 15 kGy showed a significant high ability in preserving the living cells.

## Authors’ Contributions

FA designed the study and carried out all procedures, interpreted and analyzed the data, and drafting the paper. DN monitored and evaluated all procedures and results. DD and BA contributed to material synthesis and gamma irradiation. SE contributed to selecting the non-infectious feline cadaver (donor’s bones). All authors critically reviewed, evaluated, and approved the important scientific content of the final manuscript.

## Competing Interests

The authors declare that they have no competing interests.

## Publisher’s Note

Veterinary World remains neutral about jurisdictional claims in published institutional affiliation.

## References

[1] Joshi D.O, Tank P.H, Mahida H.K, Dhami M.A, Vedpathak H.S, Karie A.S. (2010). Bone grafting: An overview. Vet. World.

[2] Fossum T.W, Dewey C.W, Horn C.V, Johnson A.L, MacPhail C.M, Radlinsky M.A.G, Schulz K.S, Willard M.D. (2013). Small Animal Surgery.

[3] Yassine K.A, Mokhtar B, Houari H, Karim A, Mohamed M. (2017). Repair of segmental radial defect with autologous bone marrow aspirate and hydroxyapatite in rabbit radius: A clinical and radiographic evaluation. Vet. World.

[4] Oryan A, Alidadi S, Moshiri A, Maffulli N. (2014). Bone regenerative medicine:Classic options, novel strategies, and future directions. J. Orthop. Surg. Res.

[5] Ghanaati S, Barbeck M, Booms P, Lorenz J, Kirkpatrick C.J, Sader R.A. (2014). Potential lack of “standardized” processing techniques for production of allogeneic and xenogeneic bone blocks for application in humans. Acta Biomater.

[6] Singh R, Singh D, Singh A. (2016). Radiation sterilization of tissue allografts: A review. World J. Radiol.

[7] Tüfekci K, Kayacan R, Kurbanoğlu C. (2014). Effects of gamma radiation sterilization and strain rate on compressive behavior of equine cortical bone. J. Mech. Behav. Biomed. Mater.

[8] Kubosch E.J, Bernstein A, Wolf L, Fretwurst T, Nelson K, Schmal H. (2016). Clinical trial and *in vitro* study comparing the efficacy of treating bony lesions with allografts versus synthetic or highly processed xenogeneic bone grafts. BMC Musculoskelet. Disord.

[9] Hangody G.M (2016). Biomechanical Analysis of Human Allografts for ACL Reconstruction, Ph.D.

[10] Nguyen H, Morgan D, Forwood M. (2007). Sterilization of allograft bone: Effects of gamma irradiation on allograft biology and biomechanics. Cell Tissue Bank.

[11] Yanke A.B, Bell R, Lee A, Kang R.W, Mather R.C, Shewman E.F, Wang V.M, Bach B.R. (2013). The biomechanical effects of 1.0 to 1.2 Mrad of γirradiation on human bone-patellar tendon-bone allografts. Am. J. Sports Med.

[12] Islam A, Chapin K, Moore E, Ford J, Rimnac C, Akkus O. (2015). Gamma radiation sterilization reduces the high-cycle fatigue life of allograft bone. Clin. Orthop. Relat. Res.

[13] Vangsness C.T, Garcia I.A., Mills C.R., Kainer M.A., Roberts M.R., Moore T.M. (2003). Allograft transplantation in the knee: Tissue regulation, procurement, processing, and sterilization. Am. J. Sports Med.

[14] Hilmy N, Abbas B, Anas F., Nather A, Yusof N, Hilmy N (2007). Validation for Processing and Irradiation of Freeze-Dried Bone Grafts. Radiation in Tissue Banking: Basic Science and Clinical Applications of Irradiated Tissue Allografts.

[15] Aguila C.M, Delcroix G.J.R, Kaimrajh D.N, Milne E.L, Temple H.T, Latta L.L. (2016). Effects of gamma irradiation on the biomechanical properties of peroneus tendons. Open Access J. Sports Med.

[16] Castell J.V, Gomez-Lechon M.J. (1997). *In vitro* Methods in Pharmaceutical Research.

[17] Zaner D.J, Yukna R.A. (1984). Particle size of periodontal bone grafting materials. J. Periodontol.

[18] American Type Culture Collection (2011). MTT Assay. http://www.atcc.org..

[19] Jaffe E.A (1987). Cell biology of endothelial cells. Hum. Pathol.

[20] Pirosa A, Gottardi R, Alexander P.G, Tuan R.S. (2018). Engineering *in vitro* stem cell-based vascularized bone models for drug screening and predictive toxicology. Stem Cell Res. Ther.

[21] Chung A.S, Ferrara N. (2011). Developmental and pathological angiogenesis. Annu. Rev. Cell. Dev. Biol.

[22] Kanczler J.M, Oreffo R.O.C. (2008). Osteogenesis and angiogenesis:The potential for engineering bone. Eur. Cell Mater.

[23] Binnington A.G, Williams W (1990). Bone remodelling and transplantation. Canine Orthopedics.

[24] Kon K, Shiota M, Ozeki M, Kasugai S. (2014). The effect of graft bone particle size on bone augmentation in a rabbit cranial vertical augmentation model: A microcomputed tomography study. Int. J. Oral Maxillofac. Implants.

[25] Wang L, Barbieri D, Zhou H, de Bruijn J.D, Bao C, Yuan H. (2015). Effect of particle size on osteoinductive potential of microstructured biphasic calcium phosphate ceramic. J. Biomed. Mater. Res. A.

[26] Hruschka V, Tang S, Ryabenkova Y, Heimel P, Barnewitz D, Möbus G, Keibl C, Ferguson J, Quadros P, Miller C, Goodchild R, Austin W, Redl H, Nau T. (2017). Comparison of nanoparticular hydroxyapatite pastes of different particle content and size in a novel scapula defect model. Sci. Rep.

[27] Burton B, Gaspar A, Josey D, Tupy J, Grynpas M, Willett T. (2014). Bone embrittlement and collagen modifications due to high-dose gamma-irradiation sterilization. Bone.

[28] Akkus O, Belaney R.M. (2005). Sterilization by gamma radiation impairs the tensile fatigue life of cortical bone by two orders of magnitude. J. Orthop. Res.

[29] Hilmy N, Febrida A, Basril A. (2000). Validation of radiation sterilization dose for lyophilized amnion and bone grafts. Cell Tissue Bank.

[30] Hangody G, Szebényi G, Abonyi B, Kiss R, Hangody L, Pap K. (2017). Does a different dose of gamma irradiation have the same effect on five different types of tendon allografts?-A biomechanical study. Int. Orthop.

[31] Harrell C.R, Djonov V, Fellabaum C, Volarevic V. (2018). Risks of using sterilization by gamma radiation:The other side of the coin. Int. J. Med. Sci.

[32] Ng K, Wanivenhaus F, Chen T, Abrams V.D, Torzili P.A, Warren R.F, Maher S.A. (2013). Differential crosslinking and radio-protective effects of geninpin on mature bovine and patella tendons. Cell Tissue Bank.

[33] International Atomic Energy Agency (2002). Specific processing procedures. The IAEA Programme in Radiation and Tissue Banking-International Standards on Tissue Banking.

[34] da Silva A.F, Antebi U, Honda E.K, Rudelli M, Guimaraes R.P. (2019). Comparative study of the osteointegration of irradiated and non-irradiated bone grafts used in patients with revision hip arthroplasty. Rev. Bras. Ortop.

[35] Yasin N.F, Ajit S.V, Saad M, Omar E. (2015). Which is the best method of sterilization for recycled bone autograft in limb salvage surgery: A radiological, biomechanical and histopathological study in rabbit. BMC Cancer.

